# The lactate-to-albumin ratio relationship with all-cause mortality in cerebral infarction patients: analysis from the MIMIC-IV database

**DOI:** 10.3389/fneur.2024.1334097

**Published:** 2024-05-01

**Authors:** Lingyan Zhao, Linna Wu, Zekun Wang, Jing Fan, Guiping Li

**Affiliations:** ^1^Wuxi Hospital of Traditional Chinese Medicine, Wuxi, China; ^2^Medicine Acupuncture and Moxibustion Department, First Teaching Hospital of Tianjin University of Traditional Chinese Medicine, Tianjin, China; ^3^National Clinical Research Center for Chinese Medicine Acupuncture and Moxibustion, Tianjin, China; ^4^School of Electrical and Information Engineering, Tianjin University, Tianjin, China

**Keywords:** lactate-to-albumin ratio, cerebral infarction, intensive care unit, MIMIC-IV database, all-cause mortality

## Abstract

**Objective:**

To examine the association of lactate-to-albumin ratio (LAR) with 30-day and 90-day mortality in patients with cerebral infarction admitted to the intensive care unit (ICU).

**Methods:**

In this retrospective observational study, 1,089 patients with cerebral infarction were recruited. The concentration of blood lactate and serum albumin on the first day of ICU admission were recorded. The relationship between LAR levels and mortality was evaluated through univariate and multivariate Cox regression analyses, four-knot multivariate restricted cubic spline regression, and Kaplan–Meier (KM) curves.

**Results:**

The overall 30-day and 90-day mortality rates in the entire cohort were 27.3 and 35.8%, respectively. KM analysis revealed a significant relationship between high LAR index and the risk of all-cause mortality (log-rank *p* < 0.001). Furthermore, multivariate Cox proportional risk analysis showed that the LAR index independently predicted the risk of 30-day mortality (HR: 1.38, 95% CI 1.15–1.64, *p* = 0.004) and 90-day mortality (HR: 1.53, 95% CI 1.32–1.77, *p* < 0.001) in the study population. Furthermore, a higher LAR exceeding 0.53 was positively correlated with the risk of 30-day and 90-day mortalities. Subsequent subgroup analyses demonstrated that LAR could predict the primary outcome.

**Conclusion:**

In summary, the LAR index is a reliable and independent predictor of increased mortality among critically ill patients suffering from cerebral infarction. Nonetheless, there is a need for additional comprehensive prospective studies to validate these findings.

## Introduction

According to the Global Burden of Disease Study 2019 report, stroke remains the second most common cause of mortality and the third most prevalent cause of disability globally (1). Cerebral infarction (CI) is the predominant form of stroke, characterized by compromised blood flow in the brain, causing tissue ischemia, hypoxia, and potentially localized necrosis (2). Although high-income nations have reported a gradual reduction in both morbidity and mortality linked to cerebral infarction in recent decades, several low-income and middle-income countries have had stagnant or even increasing rates (3). Research has shown that despite the beneficial effects of tissue plasminogen activator (tPA)-induced thrombolysis or endovascular therapy on functional outcomes in individuals with acute cerebral infarction, the overall prognosis for these patients remains unfavorable (4, 5), as evidenced by approximately 20% of patients needing intensive care unit (ICU) treatment (5). Consequently, it is imperative to identify dependable biomarkers responsible for the prognosis of patients with cerebral infarction in the ICU, thereby promoting improved patient management.

Notably, the measurement of serum lactate (Lac) level is an important indicator in clinical medicine for assessing insufficient tissue perfusion. This marker has been extensively associated with organ failure and tissue necrosis, including cerebral infarction, sepsis, pediatric critical, and trauma (6–9). An increase in lactate levels is primarily because of cellular hypoxia-ischemia, resulting in metabolic disruptions as the effective circulating volume of brain tissue diminishes. Hypoxia and energy depletion can induce injury response thereby causing poor outcomes in patients with acute cerebral infarction. Hypoalbuminemia, characterized by an adult serum albumin (Alb) level below 35 g/L, is a prevalent complication in patients with cerebral infarction. Patients admitted with concurrent hypoalbuminemia are vulnerable to infections and experience poor functional outcomes as well as high in-hospital mortality rates (10, 11). Previous research has shown that low serum Alb levels indicate recurrence and mortality in patients with cerebral infarction (12, 13). Therefore, the Alb level acts as a significant parameter in assessing the nutritional status of patients and plays a crucial role in the mortality of cerebral infarction.

In this regard, the lactate-to-albumin ratio (LAR) is a valuable indicator for assessing overall mortality in patients with cerebral infarction. We used data from the MIMIC-IV database to examine the relationship between LAR and overall mortality. In this study, we aimed to explore whether can LAR predict the prognosis of patients with CI in the ICU.

## Methods

### Study population

**Figure 1:**
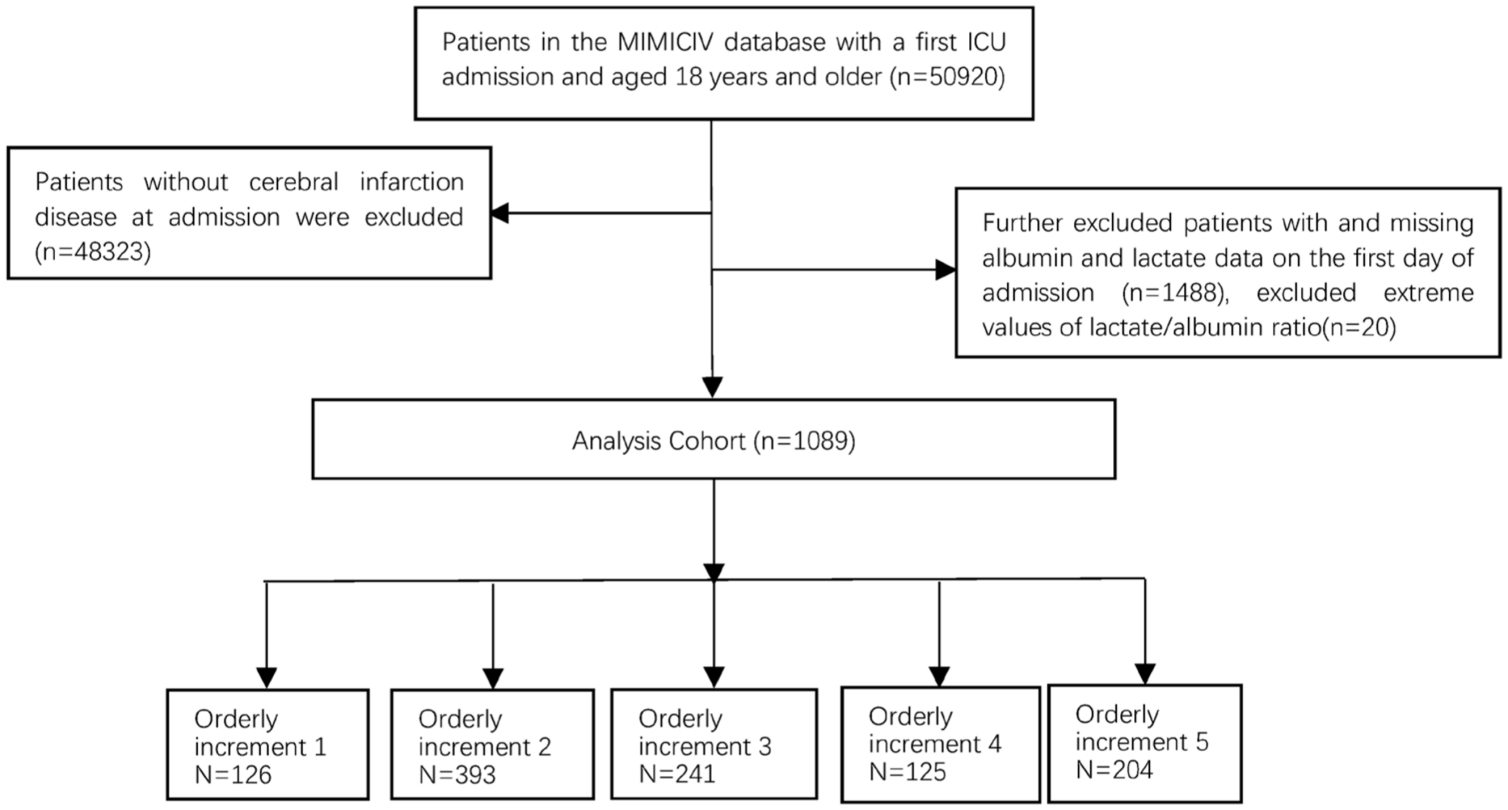
Flowchart illustrating the selection of patients from the MIMIC-IV database. MIMIC, Medical Information Mart for Intensive Care.

A retrospective cohort study was performed using data from the MIMIC-IV database between 2008 and 2019. The MIMIC database is a publicly available comprehensive and population-based critical database overseen by the Computational Physiology team at the Massachusetts Institute of Technology, which can be downloaded from.^1^ The author (Record ID 57310450), who was granted access to this database, extracted all the data. All the data used in this study were de-identified, ensuring patient anonymity. Therefore, there was no need for informed consent. We recruited participants aged 18 years or older admitted to the intensive care unit (ICU). We excluded patients who lacked albumin and lactate data on the first day of admission. Subsequently, a 1% winsorization was used for the LAR variable to minimize the impact of outliers on the accuracy of study results. Data from the initial ICU stay were considered for individuals with multiple admissions to both the ICU and hospital. This study specifically targeted individuals diagnosed with cerebral infarction based on the International Classification of Diseases, 10th edition (ICD-10, code 163) and 9th edition (ICD-9, codes 433.01, 433.11, 433.81, 433.91, 434.01, 434.11, 434.91). The study enrolled a cohort of 1,089 patients who were categorized into five groups based on the orderly increment ranges of LAR ratio on the first day of ICU admission, Includes: L1 (0.19 ≤ LAR≤0.3), L2 (0.3 < LAR≤0.5), L3 (0.5 < LAR≤0.7), L4 (0.7 < LAR≤0.9), L5 (0.9 < LAR≤4.16). Figure 1 shows the flowchart of patient screening.

### Data collection

Data were extracted from the MIMIC-IV database using PostgreSQL (version 11.21) and structured query language (SQL). The code of demographic data, comorbidities, and severity score were acquired from the GitHub website.^2^ The following data were collected: (1) Demographics: age, gender, race, height, weight, and other general demographics; (2) Comorbidities included diabetes, hypertension, heart failure, Atrial fibrillation and sepsis; (3) The laboratory results extracted were from first-day inspection after admission to the ICU, including Serum sodium, Serum potassium, Creatinine, Chloride, blood urea nitrogen, Hemoglobin, white blood cell, red blood cell, platelet count, prothrombin time, Glucose, Albumin, and Lactate; (4) SOFA sequential organ failure assessment, SAPSII simplified acute physiological score II and Glasgow Coma Scale score were also included.

Missing values were assumed to be randomly missing. Variables with missing values exceeding 20% were excluded from the univariate analysis. The multiple imputation method was used to impute variables with missing values below 20%. All the included variables had less than 20% missing values.

### Primary outcome

The primary outcomes examined were all-cause 30-day mortality and 90-day mortality, whereas the secondary outcome assessed all-cause mortality within a follow-up period of 180 days.

### Data analysis

The Shapiro–Wilk test was used to assess continuous variables, which were presented as mean ± SD if they adhered to a normal distribution, or as median [interquartile range (IQR)] if they did not. The comparison of continuous variables was conducted using either the Student T-test or the Mann–Whitney test, based on their distribution. Categorical variables were represented by frequencies with percentages. The evaluation of significant differences was performed using either the Pearson Chi-square test or Fisher’s exact test.

The continuous variables of age and weight were transformed into categorical variables, specifically age (≤70 years and > 70 years) and weight (≤80 kg and > 80 kg). Kaplan–Meier survival analysis was performed to assess the relationship between LAR and overall mortality, assessing the differences using the log-rank test. Additionally, multivariable Cox proportional hazard regression analysis was used to evaluate the effect of LAR on the overall mortality of individuals with cerebral infarctions, estimating hazard ratios (HRs) and 95% confidence intervals (CIs). Variables with a *p*-value less than 0.2 in the univariate Cox analysis were incorporated into the multivariate Cox regression analyses. We excluded variables with a variance inflation factor (VIF) greater than 5. The model included LAR as both continuous and categorical variables, with the lowest level (L1) acting as the reference, p for trends was also calculated. Ultimately, a multivariate model was used to analyze clinically significant and prognostic-related factors. Model 1 without any adjustments. In model 2, adjustments were made for age, race, and Sepsis. Model 3 included adjustments for age, race, blood urea nitrogen, white blood cells, platelets, and Sepsis. The assumption of proportional hazards in the Cox proportional hazards regression models was evaluated using the Proportion Hazards Assumption test.

Exploratory restricted cubic spline (RCS) Cox regression was used to investigate the presence of a potential non-linear relationship between the rate of LAR and adverse perinatal outcomes, including 30-day and 90-day mortalities, among individuals diagnosed with cerebral infarction. Further, interaction tests and stratified analyses were conducted to validate the consistency of the prognostic value of the LAR for major outcomes, considering age (≤70 and > 70 years), SOFA score (<5 and ≥ 5), Heart failure (yes and no), Atrial fibrillation (yes and no), and Sepsis (yes and no). All statistical analyses were performed using Stata (17.0, IBM) and R (version 4.3.1, Austria) software. All analyses were two-tailed. A *p*-value < 0.05 was considered statistically significant.

## Results

The present study enrolled 1,089 participants, with an average age of 68.39 among patients diagnosed with cerebral infarction; 571 (52.43%) were males and 518 (47.57%) were females. The mean value of LAR was 0.68 (0.37, 0.78).

### Baseline characteristics of cerebral infarction patients

All the participants were categorized into two cohorts based on their survival outcome within 30 days. Table 1 shows the fundamental attributes of these cohorts. Patients in the non-surviving cohort were of advanced age and had a greater probability of developing sepsis (*p* < 0.05). Regarding laboratory indicators, the non-surviving cohort had significantly higher levels of white blood cell count, serum sodium, serum potassium, serum chlorine, blood glucose, serum creatinine, blood urea nitrogen, and blood lactate compared to the surviving cohort (*p* < 0.05). In contrast, the groups that survived had significantly higher levels of red blood cells (RBC), hemoglobin (Hb), and albumin, unlike the groups that did not survive (*p* < 0.05). The non-survival group had a considerably increased LAR unlike the survival group (*p* < 0.001).

**Table 1:** Comparisons of baseline characteristics between survivors and non-survivors.

Characteristic	Overall (*N* = 1,089)	Survivors (*N* = 792)	Non-survivors (*N* = 297)	*p*-value
Age, years	68.39 (57.88, 80.62)	66.98 (56.60, 79.19)	72.17 (62.58, 83.68)	<0.001
Weight, kg	80.52 (65.8, 91)	81.50 (66.85, 91.9)	77.89 (63.2, 90)	0.0421
Male, *n* (%)	571 (52.43)	417 (57.20)	155 (52.19)	0.55
Race
1. Other	226	127	99	<0.001
2. Asian	35	26	9	
3. Black	141	110	31	
4. White	647	496	151	
5. Hispanic	40	33	7	
SAPSII score	38.85 (29, 47)	35.97 (27, 43)	46.53 (37, 56)	<0.001
SOFA score	6.36 (4, 8)	5.75 (3, 7)	7.98 (5, 11)	<0.001
GCS score	12.57 (11, 15)	12.77 (11, 15)	12.03 (10, 15)	0.72
Laboratory tests	
Serum sodium, mEq/L	134.54 (132, 138)	134.00 (132, 138)	135.96 (133, 140)	<0.001
Serum potassium, mEq/L	3.39 (3, 3.7)	3.35 (3, 3.6)	3.48 (3.1, 3.8)	0.0064
Creatinine mg/dL	0.96 (0.6, 1)	0.90 (0.5, 1)	1.13 (0.6, 1.3)	<0.001
Chloride, mEq/L	98.31 (95, 102)	97.54 (95, 101)	100.36 (96, 105)	<0.001
BUN, mg/dL	16.54 (9, 20)	14.35 (9, 17)	22.38 (12, 28)	<0.001
Hemoglobin, g/dL	12.20 (10.7, 13.5)	12.31 (10.9, 13.6)	11.89 (10.3, 13.4)	0.0031
WBC, K/uL	7.78 (5.6, 9.4)	7.06 (5.5, 8.5)	9.71 (6.2, 12.3)	<0.001
RBC, K/uL	4.03 (3.55, 4.47)	4.06 (3.6, 4.5)	3.95 (3.4, 4.42)	0.0073
platelet count, K/uL	327.04 (215, 415)	344.9 (228, 431)	279.42 (176, 363)	<0.001
PT	12.72 (11.4, 13.2)	12.46 (11.3, 13)	13.42 (11.8, 14.1)	<0.001
Glucose, mg/dL	150.92 (106, 173)	147.15 (104, 167)	161.96 (115, 190)	<0.001
Albumin, g/dL	3.36 (2.9, 3.9)	3.41 (2.9, 4)	3.21 (2.7, 3.7)	<0.001
Lactate, mmol/L	2.18 (1.3, 2.5)	2.04 (1.2, 2.35)	2.54 (1.3, 3)	<0.001
LAR	0.68 (0.37, 0.78)	0.63 (0.36, 0.70)	0.82 (0.43, 0.94)	<0.001
Comorbidities, *n* (%)	
Sepsis	624 (57.30)	417 (52.65)	207 (69.70)	<0.001
Hypertension	523 (48.03)	373 (47.10)	150 (50.50)	0.316
Heart failure	312 (28.65)	218 (27.53)	94 (31.65)	0.18
Atrial fibrillation	421 (38.66)	300 (37.88)	128 (43.10)	0.116
Diabetes	356 (32.69)	262 (33.08)	94 (31.65)	0.654

Survivors and non-survivors were based on 30-day mortality, Values are number (percentage), or median (25th-75th percentile). SOFA, sequential organ failure assessment; SAPSII, simplified acute physiological score II; GCS, Glasgow Coma Scale; BUN, blood urea nitrogen; WBC, white blood cell; RBC, red blood cell; PT, prothrombin time; LAR, lactate to albumin ratio.

### Incidence of all-cause mortality among different groups

**Figure 2:**
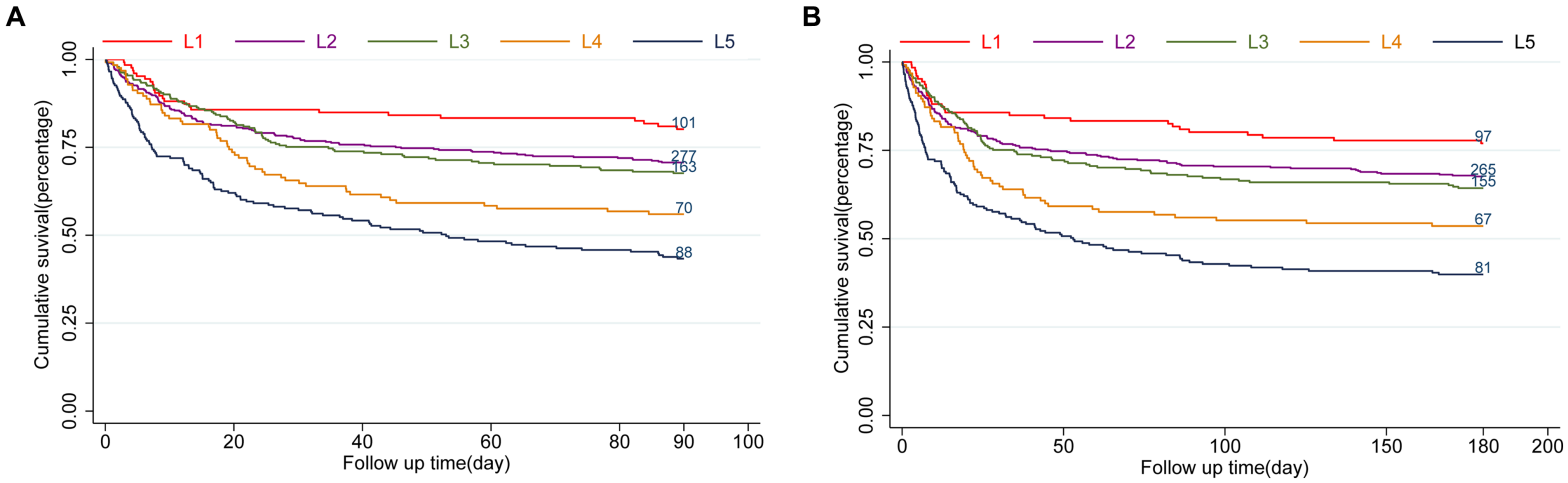
Kaplan–Meier survival analysis curves for all-cause mortality. LAR orderly increment: L1 (0.19 ≤ LAR≤0.3), L2 (0.3<LAR≤0.5), L3 (0.5<LAR≤0.7), L4 (0.7<LAR≤0.9), L5 (0.9<LAR≤4.16). Kaplan–Meier curves showing cumulative probability of all-cause mortality according to groups at 90 days **(A)**, and 180 days **(B)**.

The LAR ratio was divided into five groups based on increasing ranges in an orderly manner: L1 (0.19 ≤ LAR≤0.3), L2 (0.3 < LAR≤0.5), L3 (0.5 < LAR≤0.7), L4 (0.7 < LAR≤0.9), L5 (0.9 < LAR≤4.16). The patient survival in all groups was presented in Kaplan–Meier curves, indicating a statistically significant difference in survival rate between groups at 90 days (L1: 80.2% vs. L2: 70.5% vs. L3: 67.6% vs. L4: 56% vs. L5: 43.1%, log-rank *p* = 0.021, Figure 2A). Similarly, a significant outcome was observed during the 180-day follow-up period (L1: 77.0% vs. L2: 67.4% vs. L3: 64.3% vs. L4: 53.6% vs. L5: 39.7%, log-rank *p* = 0.021, Figure 2B).

A linear association between LAR and all-cause mortality.

**Figure 3:**
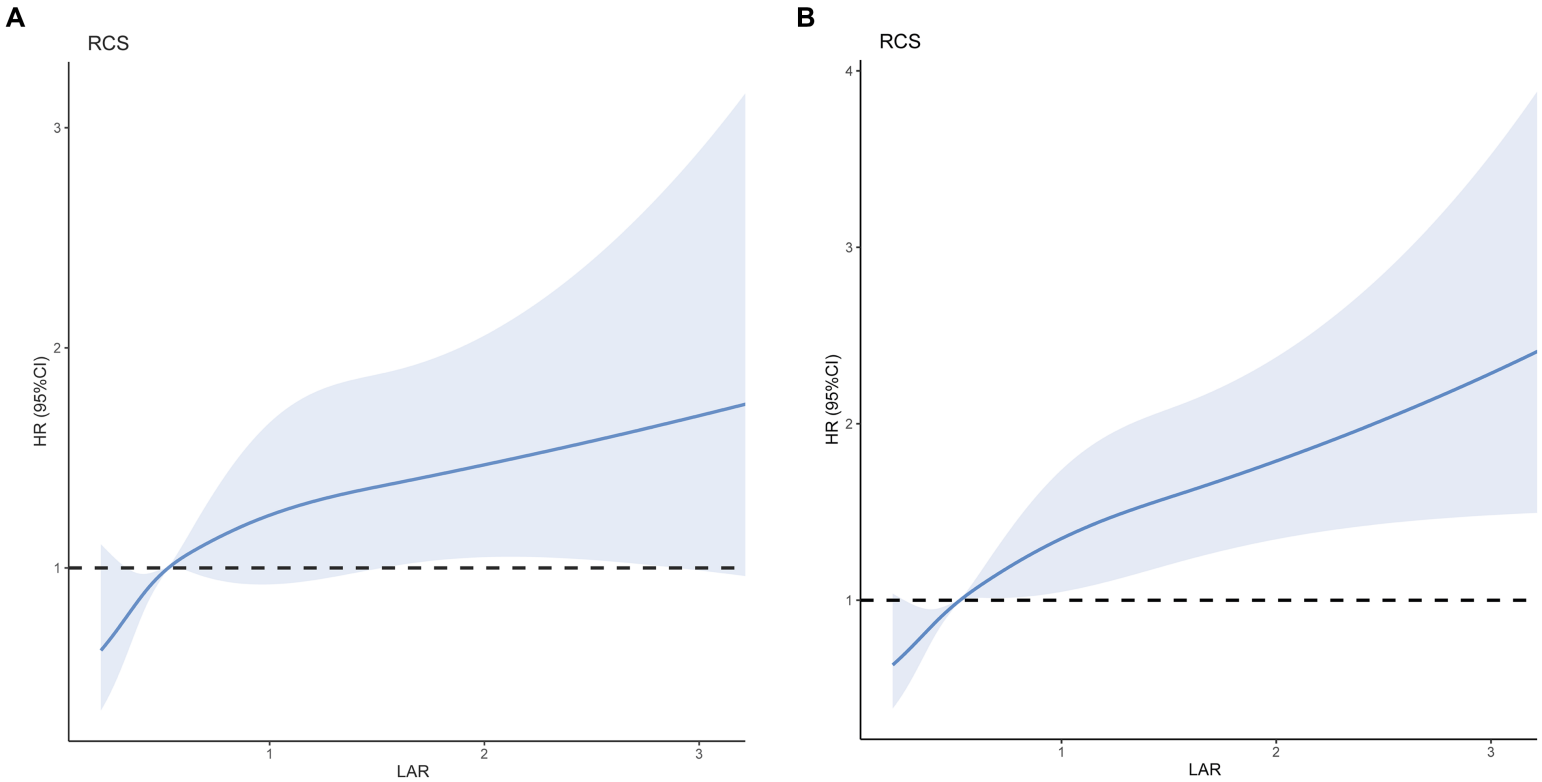
Multivariable RCS regression showed the linear association between the LAR and 30-day **(A)** and 90-day **(B)** mortality afteradjusted for age, race, blood urea nitrogen, white blood cells, platelets, and Sepsis. The solid line depicts the estimated values, while the blue area represents their corresponding 95% confidence intervals (CIs). The dotted horizontal lines show the 1.0 hazard ratio. HR, hazard ratio; CI, confdence interval; LAR, lactate/albumin ratio; RCS, restricted cubic spline.

After adjusting for age, race, blood urea nitrogen, white blood cells, platelets, and sepsis (Figure 3A), RCS models were used to further investigate the potential linear relationship between LAR (as a categorical variable) and the risk of 30-day mortality (*p* for non-linearity = 0.1465). Furthermore, we assessed the potential linear relationship between LAR and 90-day fatality (*p* value for non-linearity = 0.0716), at the same time adjusting for age, race, blood urea nitrogen, white blood cells, platelets, and sepsis (Figure 3B). When the LAR exceeded 0.53, we noted a positive correlation with the risk of 30-day and 90-day mortality in individuals suffering from cerebral infarction. Additionally, the HR approached 1 and the cutoff value remained at 0.53.

### LAR level and all-cause mortality

In the initial step, we preformed COX univariate analysis in which variables with a significance level of *p* < 0.2 were included in the subsequent COX multivariate analysis. Additionally, we systematically eliminated variables with Variance Inflation Factor (VIF) values exceeding 5 individually (14). The detailed procedure for this screening process is presented in Supplementary Tables S1 and S2. The Cox proportional risk analysis revealed a significant relationship between the LAR and 30-day mortality. This relationship was observed upon using LAR as a continuous variable in both crude Cox models (HR, 1.61 [95% CI 1.37–1.89], *p* < 0.001) and multivariate Cox models (HR, 1.38 [95% CI 1.15–1.64], *p* = 0.004). When the LAR was treated as a categorical variable, the crude Cox model displayed varying levels of risk across different categories (L1 vs. L2: HR, 1.64 [95% CI 0.99–2.72] *p* = 0.0.0561; L3: HR, 1.78 [95% CI 1.05–3.02] *p* = 0.031; L4: HR, 2.60 [95% CI 1.50–4.51] *p* = 0.001; L5: HR, 3.64 [95% CI 2.19–6.05] *p* < 0.001 *p* for trend <0.001). These relationships remained statistically significant even after adjusting for confounding factors in fully adjusted models (L1 vs. L2: 95% CI HR, 1.47 [95% CI 0.88–2.45] *p* = 0.137; L3: HR, 1.67 [95% CI 0.98–2.84] *p* = 0.057; L4: HR, 2.14 [95% CI 1.23–3.73] *p* = 0.007; L5: HR, 2.23 [95% CI 1.33–3.76] *p* = 0.0032 *p* for trend <0.001). The test for the proportional hazard assumption yielded a non-significant result (*p* = 0.2481). Additionally, we obtained similar findings when conducting multivariate Cox proportional risk analysis to assess the relationship between the LAR and 90-day mortality (Table 2). Specifically, the proportional hazard assumption remained valid when considering LAR as categorical variables in fully adjusted models (*p* = 0.115).

**Table 2:** Cox proportional hazard ratios (HR) for 30-day mortality and 90-day mortality.

Categories	Model 1	Model 2	Model 3
	HR (95%)	*p*-value	*p* for trend	HR (95%)	*p*-value	*p* for trend	HR (95%)	*p*-value	*p* for trend
30-day mortality	
Continuous variable per	1.61 (1.37–1.89)	<0.001		1.65 (1.27–2.13)	<0.001		1.38 (1.15–1.64)	0.004	
1 unit
Orderly increment	
L1 (*N* = 126)			<0.001			<0.001			<0.001
L2 (*N* = 393)	1.64 (0.99–2.72)	0.056		1.51 (0.91–2.51)	0.113		1.47 (0.88–2.45)	0.137	
L3 (*N* = 241)	1.78 (1.05–3.02)	0.031		1.60 (0.94–2.72)	0.081		1.67 (0.98–2.84)	0.057	
L4 (*N* = 125)	2.60 (1.50–4.51)	0.001		2.48 (1.43–4.32)	0.001		2.14 (1.23–3.73)	0.007	
L5 (*N* = 204)	3.64 (2.19–6.05)	<0.001		3.02 (1.80–5.04)	<0.001		2.23 (1.33–3.76)	0.003	
90-day mortality	
Continuous variable per	1.71 (1.49–1.95)	<0.001		1.63 (1.41–1.87)	<0.001		1.53 (1.32–1.77)	<0.001	
1 unit
Orderly increment	
L1 (*N* = 126)			<0.001			<0.001			0.001
L2 (*N* = 393)	1.58 (1.02–2.43)	0.039		1.43 (0.93–2.22)	0.104		1.44 (0.93–2.23)	0.098	
L3 (*N* = 241)	1.73 (1.10–2.71)	0.017		1.54 (0.98–2.42)	0.063		1.63 (1.04–2.56)	0.035	
L4 (*N* = 125)	2.56 (1.59–4.10)	<0.001		2.40 (1.49–3.87)	<0.001		2.04 (1.36–3.52)	0.001	
L5 (*N* = 204)	3.78 (2.45–5.82)	<0.001		3.11 (2.01–4.83)	<0.001		2.57 (1.65–3.99)	<0.001	

Cox proportional hazards models were employed to estimate HR and 95% CI. Model 1, unadjusted; Model 2, adjusted for age, race, and Sepsis; Model 3, adjusted for age, race, blood urea nitrogen, white blood cells, platelets, and Sepsis. LAR, Orderly increment: L1 (0.19 ≤ LAR ≤ 0.3), L2 (0.3<LAR ≤ 0.5), L3 (0.5<LAR ≤ 0.7), L4 (0.7<LAR ≤ 0.9), L5 (0.9<LAR ≤ 4.16).

### Subgroup analysis

The association between the LAR level and all-cause mortality was examined in various subgroups of the study population, including age, sepsis, heart failure, atrial fibrillation, and SOFA score. The relationship between the LAR ratio and the 90-day mortality in patients with critical cerebral infarction was assessed, with subgroup analyses conducted for age, sepsis, heart failure, atrial fibrillation, and SOFA score. The results of all subgroup analyses were consistent and indicated a significant association between the LAR ratio and 90-day mortality (all *p* < 0.05), with no interactions observed (all *p* for interaction>0.05). Similar findings were noted when conducting stratified analyses of the LAR level and 30-day mortality. However, no statistically significant differences were found in subgroups of individuals aged ≥70 years (HR, 1.27 [95% CI 0.95–1.69]), with SOFA scores ≤5 (HR, 1.23 [95% CI 0.78–1.93]), and without sepsis (HR, 1.55 [95% CI 0.98–2.45]) (all *p* > 0.05) in relation to 30-day mortality. Table 3 shows that data.

**Table 3:** The subgroup analysis results of the multivariable-adjusted HR for the association between the LAR and 30-day and 90-day mortality.

Subgroup analysis		Model 1	Model 2			Model 3		
	*N*		OR	*p*-value	*p* for interaction	OR	*p*-value	*p* for interaction
30-day mortality	
AGE, year				0.419				0.425
≥70	556	ref	1.42 (1.10–1.83)	0.007		1.27 (0.95–1.69)	0.11	
<70	553	ref	1.57 (1.26–1.96)	<0.001		1.42 (1.13–1.77)	0.003	
SOFA					0.854			0.66
>5	615	ref	1.49 (0.98–2.25)	0.062		1.35 (1.10–1.66)	0.005	
≤5	474	ref	1.49 (1.24–1.79)	<0.001		1.23 (0.78–1.93)	0.383	
Sepsis					0.094			0.208
Yes	624	ref	1.46 (1.21–1.75)	<0.001		1.34 (1.10–1.62)	0.004	
No	465	ref	2.0 (1.38–2.90)	<0.001		1.55 (0.98–2.45)	0.06	
Heart failure					0.55			0.36
Yes	312		1.74 (1.33–2.27)	<0.001		1.54 (1.13–2.10)	0.007	
No	777		1.56 (1.27–1.90)	<0.001		1.31 (1.05–1.63)	0.015	
Atrial fibrillation					0.059			0.921
Yes	428		2.04 (1.56–2.67)	<0.001		1.52 (1.12–2.07)	0.007	
No	661		1.46 (1.18–1.80)	0.001		1.39 (1.11–1.74)	0.004	
90-day mortality	
AGE, year					0.893			0.963
≥70	556	ref	1.60 (1.31–1.96)	<0.001		1.52 (1.21–1.90)	<0.001	
<70	553	ref	1.59 (1.31–1.93)	<0.001		1.48 (1.22–1.80)	<0.001	
SOFA					0.3			0.842
>5	615	ref	1.54 (1.32–1.80)	<0.001		1.48 (1.24–1.76)	<0.001	
≤5	474	ref	1.77 (1.27–2.47)	0.01		1.57 (1.10–2.22)	0.012	
Sepsis3					0.09			0.12
Yes	624	ref	1.56 (1.33–1.82)	<0.001		1.47 (1.25–1.72)	<0.001	
No	465	ref	2.05 (1.49–2.80)	<0.001		1.69 (1.17–2.44)	0.005	
Heart failure					0.426			0.266
Yes	312		1.86 (1.49–2.32)	<0.001		1.76 (1.37–2.26)	<0.001	
No	777		1.64 (1.39–1.95)	<0.001		1.43 (1.19–1.72)	<0.001	
Atrial fbrillation					0.132			0.942
Yes	428		2.05 (1.61–2.61)	<0.001		1.62 (1.24–2.12)	<0.001	
No	661		1.62 (1.37–1.92)	<0.001		1.55 (1.30–1.86)	<0.001	

Model 2, adjusted for age, race, and Sepsis; Model 3, adjusted for age, race, blood urea nitrogen, white blood cells, platelets, and Sepsis.

## Discussion

Stroke is the second most common cause of mortality across the globe, with the highest rates of both fatality and morbidity among all diseases, after premature death and disability arising from illness (1, 15). Cerebral infarction, the primary manifestation of stroke, accounts for most of the fatalities and impairments globally. Furthermore, approximately 20% of individuals afflicted with cerebral infarction require intensive care intervention (5). This study provides evidence of a significant effect of the lactate-to-albumin ratio (LAR) as an independent determinant of mortality rates in patients with cerebral infarction, both within 30 days and 90 days. The findings revealed a consistent increase in mortality rates within these timeframes as LAR increases, with a statistically significant trend (*p*-value for trend = 0). As the ratio increased, the distinction became more discernible, and a positive correlation was observed between the risk of mortality within 30 days, 90 days, and the LAR, but solely when the LAR exceeded 0.53. In this study, the fully adjusted model showed that the LAR, as an ordinal variable, confirms the Cox proportional risk assumption with a p-value exceeding 0.5. Furthermore, our viewpoint is strengthened by subgroup analyses.

In recent years, researchers have increasingly studied the association of inflammation with outcomes of patients with cerebral infarction. Among patients with cerebral infarction, the relationship between the distribution width of red blood cells in peripheral blood (RDW) and human serum albumin (Alb) acts as a dependable indicator for acute ischemic stroke (AIS) and a distinct predictor for all-cause mortality within 30 days (16). The clinical assessment of 30-day mortality in patients with ischemic stroke or hemorrhagic stroke can be conducted using various ratios, including neutrophil/lymphocyte ratio (NLR), platelet/lymphocyte ratio, neutrophil/albumin ratio, prognostic nutritional index (PNI), systemic immune inflammation index (SII), and red cell distribution width/albumin ratio (RA) (17–19). In our research, the LAR, which is an innovative predictor of inflammation, holds significant clinical importance in evaluating the risk of death from any cause among individuals with cerebral infarction.

The presence of lactate (Lac) in the body indicates inadequate tissue perfusion and cellular hypoxia due to anaerobic metabolism. In the absence of adequate oxygen and tissue perfusion, lactate dehydrogenase suppresses the breakdown of pyruvate, resulting in lactate accumulation. Previous studies have shown that individuals with brain injury have increased lactate levels (8). Within the scope of our study, we observed significantly increased lactate levels in patients with cerebral infarction who did not survive, unlike those who survived. These results align with previous research on critically ill patients (20, 21). Moreover, lactate is a marker for organ failure and mortality. Nonetheless, alternative studies have shown that in severe cases, patients may present reduced levels of lactate in their venous bloodstream, thereby compromising the reliability of using lactate as the sole predictor of patient prognosis (22). Albumin, a key nutrient primarily synthesized by the liver, plays a critical role in stabilizing the colloid osmotic pressure of plasma and acts as an important means of material transportation within the bloodstream (23). Furthermore, the serum Alb level is influenced by the nutritional status of patients and is closely associated with the inflammatory response. The presence of Alb causes the synthesis of anti-inflammatory molecules including lipoxins, lysins, and protections, thus promoting wound healing and limiting disease progression (24). This process involves a significant consumption of Alb, and the extent of its decline directly correlates with the inflammation degree (25, 26). Previous studies have shown that Alb can act as an independent prognostic factor for ischemic stroke (13, 27). Furthermore, our findings confirmed that Alb levels significantly decreased in the cohort of cerebral infarction patients who did not survive. However, the concentration of serum albumin can be influenced by various factors, including inflammation, nutritional support, and chronic illness. Consequently, its predictive value in a single assessment may be limited. The use of the serum lactate to serum Alb ratio (LAR) can improve the accuracy of prognosticating outcomes in patients with cerebral infarction by counterbalancing the effect of a singular factor on regulatory mechanisms through the contrasting effects induced by two separate mechanisms.

Several studies have demonstrated that oxidative stress, inflammatory damage, and immune injury contribute to the onset and progression of cerebral infarction (28–30). The inflammatory and oxidative stress conditions within the body have been found to directly trigger abnormal glycolysis, resulting in the accumulation of lactic acid and excessive glycogen consumption. This increases the production of lactic acid during the biological aging process (31, 32). Lactic acid is generated as a result of immune activation, and may stimulate pro-inflammatory and immune-regulating processes (33). In addition, endogenous lactic acid serves a neuroprotective function by mitigating excitotoxicity, oxidative stress, and traumatic injury (34–36). This potentially provides a mechanism by which lactate is generated during cerebral infarction, suggesting its impact on the prognosis of patients. Research has demonstrated that serum Alb not only signifies the nutritional status of the individual but also correlates with the extent of inflammatory response and oxidative stress, with decreased Alb levels indicating heightened oxidative stress and inflammation (37, 38). In addition, Alb can reduce blood viscosity and improve arterial reactivity, thereby mitigating the detrimental effects of ischemia–reperfusion injury (39, 40). A previous study found that Alb, acting as an antithrombotic agent, reduces the levels of multiple coagulation factors, destabilizes blood clots, and prevents platelet aggregation, thereby influencing thrombosis (41). Consequently, it may affect the prognosis of patients with cerebral infarction.

LAR has been used in prognostication of overall mortality in patients diagnosed with sepsis, acute cardiac insufficiency, respiratory insufficiency, and acute pancreatitis-induced inflammation (42–44). Research has shown a significant relationship between increased LAR values in the early stages of the disease and an augmented likelihood of concurrent organ dysfunction (45). Infections related to stroke can potentially cause sepsis, which is characterized by a disruption of host response to infection, subsequently causing organ impairment and an increased mortality rate (46). The research findings indicate a correlation between sepsis and cerebral infarction (CI), suggesting that sepsis increases the probability of developing CI and is linked to increased negative outcomes and mortality rates in patients with CI (47–50). Subgroup analyses revealed no significant interaction between the subgroups sepsis, heart failure, and atrial fibrillation. This indicates that the LAR acts as a dependable indicator of all-cause mortality in the entire population. Moreover, within the realm of medical practice, the SOFA scale assumes a pivotal function in sepsis detection (51) acting as an appropriate tool for prognosticating mortality rate among individuals with severe acute cerebral infarction (52). We classified the subjects based on their sofa score to mitigate the effect on outcome prediction. No correlation was observed between the two subcategories. Although no interaction was observed among all subgroups, specific analyses showed inconsistent *p* values within the two subgroups, yet consistently exceeding 1 on hazard ratios. These discrepancies may be attributed to the limited sample size and survival data. Consequently, future studies with a substantial number of participants are necessary to verify our findings.

### Limitations

This work provides evidence that LAR is an independent predictor of 30-day and 90-day mortality in patients with cerebral infarction. However, it has compelling limitations. First, despite conducting subgroup analyses and using multivariable-adjusted analyses, there is a possibility of selection bias and limited sample size. Secondly, the LAR may be influenced by several factors. For instance, shock, sepsis, and poor systemic or local tissue perfusion may alter the lactate (lac) level, as well as other factors such as comorbidities with alcoholism, severe hepatitis, diabetes mellitus, severe asthma, malignant tumors, underlying diseases, use of medications such as biguanides, catecholamines, and other drugs that can increase the concentration of lactate (53–55). In addition to inflammation, nutritional support, liver function, cardiovascular disease, viral infections, renal disease, neoplasms, and auto-rheumatic immune disorders can potentially lower albumin levels (56). Moreover, this analysis mainly focused on the prognostic significance of the LAR at the initial day of ICU admission in patients with cerebral infarction. However, it is possible that the LAR may have continuously fluctuated during the follow-up period. Thus, failure to analyze the potential effect of dynamic changes of LAR on patient outcomes may be a limitation of our study. This was a retrospective study conducted in a single center. Thus, rigorous prospective and multicenter studies are required to validate our findings.

## Conclusion

In conclusion, this is the first study to establish a correlation between the LAR and both 30-day and 90-day mortality rates within a cohort of individuals with cerebral infarction. Our findings indicate that an increased LAR acts as an autonomous prognostic indicator for overall mortality in patients with cerebral infarction. This relationship remains statistically significant even after accounting for potential confounding factors. Further analyses are advocated to further confirm the association between the LAR and all-cause mortality among critically ill patients with cerebral infarction.

## Data availability statement

The datasets presented in this study can be found in online repositories. The names of the repository/repositories and accession number(s) can be found below: The data utilized in this study is derived from the MIMIC database, a publicly accessible and extensive critical database managed by the Computational Physiology team at the Massachusetts Institute of Technology. The database can be obtained from the following link: (https://physionet.org/content/mimiciv/2.2/).

## Ethics statement

The requirement of ethical approval was waived by the review committee of Massachusetts Institute of Technology and Beth Israel Deaconess Medical Center. For the studies involving humans because this study adhered to the principles outlined in the Declaration of Helsinki. Approval for utilizing the MIMIC-IV database was granted by the review committee of Massachusetts Institute of Technology and Beth Israel Deaconess Medical Center. As the data is publicly accessible within the MIMIC-IV database, the study does not necessitate an ethical approval statement. The studies were conducted in accordance with the local legislation and institutional requirements. The ethics committee/institutional review board also waived the requirement of written informed consent for participation from the participants or the participants’ legal guardians/next of kin because as the data is publicly accessible within the MIMIC-IV database, the study does not necessitate an informed consent. Written informed consent was not obtained from the individual(s) for the publication of any potentially identifiable images or data included in this article because The data utilized in this study is derived from the publicly accessible MIMIC database, wherein all information is de-identified, thereby obviating the necessity of obtaining informed consent.

## Author contributions

LZ: Data curation, Formal analysis, Methodology, Writing – original draft, Writing – review & editing. LW: Supervision, Writing – original draft. ZW: Formal analysis, Methodology, Visualization, Writing – review & editing. JF: Data curation, Writing – review & editing. GL: Supervision, Writing – review & editing.

## Footnotes

^1^ https://physionet.org/content/mimiciv/2.2/

^2^ https://github.com/MIT-LCP/mimic-code
