# Supplementary material for: The lactate-to-albumin ratio relationship with all-cause mortality in cerebral infarction patients: analysis from the MIMIC-IV database
Source: Front Neurol. 2024 May 1;15:1334097. doi: 10.3389/fneur.2024.1334097 (PMC11110838; doi:10.3389/fneur.2024.1334097)
Supplement: Supplementary file 1 [file Table_1.DOCX]

Table S1 P value calculated by univariate Cox proportional hazard regression

| Characteristic | HR | p-value | [95% conf. interval] | |
| --- | --- | --- | --- | --- |
| Age， years | 1.52641 | 0 | 1.210321 | 1.925048 |
| Weight， kg | 0.79701 | 0.055 | 0.632152 | 1.004862 |
| male | 0.950861 | 0.665 | 0.756749 | 1.194764 |
| GCS score | 0.942751 | 0 | 0.913413 | 0.973031 |
| SAPSII score | 1.042604 | 0 | 1.035085 | 1.050178 |
| SOFA score | 1.111661 | 0 | 1.08476 | 1.139229 |
| Serum sodium, mEq/L | 1.076165 | 0 | 1.049452 | 1.103557 |
| Serum potassium, mEq/L | 1.689021 | 0 | 1.346815 | 2.118176 |
| Chloride, mEq/L | 1.093477 | 0 | 1.069632 | 1.117853 |
| Creatinine mg/dL | 1.187325 | 0 | 1.095996 | 1.286264 |
| Hemoglobin, g/dL | 0.914886 | 0.003 | 0.862865 | 0.970043 |
| BUN, mg/dL | 1.039877 | 0 | 1.03296 | 1.046841 |
| RBC, K/uL | 0.806467 | 0.015 | 0.677887 | 0.959436 |
| WBC, K/uL | 1.120156 | 0 | 1.100785 | 1.139869 |
| platelet count, K/uL | 0.997259 | 0 | 0.996399 | 0.998119 |
| PT | 1.136047 | 0 | 1.094901 | 1.178739 |
| Glucose, mg/dL | 1.002174 | 0.002 | 1.000792 | 1.003558 |
| Albumin, g/dL | 0.735813 | 0 | 0.628042 | 0.862077 |
| Lactate, mmol/L | 1.146004 | 0 | 1.088916 | 1.206084 |
| LAR | 1.611177 | 0 | 1.374494 | 1.888615 |
| sepsis | 1.81118 | 0 | 1.412289 | 2.322734 |
| Hypertension | 1.15136 | 0.226 | 0.916375 | 1.446603 |
| Atrial fibrillation | 1.187905 | 0.143 | 0.943388 | 1.495797 |
| Heart failure | 1.14531 | 0.279 | 0.89589 | 1.464171 |
| diabetes | 0.940454 | 0.623 | 0.736166 | 1.201433 |
| race | 0.767064 | 0 | 0.705313 | 0.834221 |

Table S2 the variance inflation factor (VIF) was calculated in the multivariable Cox regression analysis.

| Characteristic | VIF1 | VIF2 | VIF3 | VIF4 | VIF5 | p-value5 | VIF6 | p-value6 |
| --- | --- | --- | --- | --- | --- | --- | --- | --- |
| Age， years | 3.1 | 3.08 | 2.95 | 2.47 | 2.44 | 0.007 | 2.04 | 0.002 |
| Weight， kg | 2.08 | 2.07 | 1.94 | 1.89 | 1.84 | 0.263 | / |  |
| GCS score | 19.57 | 16.93 | 14.58 | / |  |  |  |  |
| SAPSII score | 20.72 | 19.11 | / |  |  |  |  |  |
| SOFA score | 7.94 | 7.85 | 7.46 | 5.54 | / |  |  |  |
| Serum sodium, mEq/L | 805.97 | / |  |  |  |  |  |  |
| Serum potassium, mEq/L | 62.82 | 43.2 | / |  |  |  |  |  |
| Chloride, mEq/L | 620.15 | / |  |  |  |  |  |  |
| Creatinine mg/dL | 3.74 | 3.62 | 3.49 | 3.49 | / |  |  |  |
| Hemoglobin, g/dL | 101.19 | 30.47 | / |  |  |  |  |  |
| BUN, mg/dL | 6.12 | 5.98 | 5.52 | 5.31 | 3.24 | <0.001 | 3.15 | <0.001 |
| RBC, K/uL | 105.94 | / |  |  |  |  |  |  |
| WBC, K/uL | 5.71 | 5.65 | 4.95 | 4.68 | 4.68 | <0.001 | 4.59 | <0.001 |
| platelet count, K/uL | 6.2 | 5.88 | 4.92 | 4.67 | 4.61 | <0.001 | 4.51 | <0.001 |
| PT | 37.61 | 32.31 | / |  |  |  |  |  |
| Glucose, mg/dL | 6.35 | 6.31 | 5.91 | / |  |  |  |  |
| Albumin, g/dL | 72.75 | / |  |  |  |  |  |  |
| Lactate, mmol/L | 45.9 | 21.05 | / |  |  |  |  |  |
| LAR | 45.35 | 21.05 | 3.09 | 2.94 | 2.7 | 0.001 | 2.7 | <0.001 |
| sepsis | 3.52 | 3.48 | 3.42 | 3.37 | 2.48 | <0.001 | 2.46 | <0.001 |
| Atrial fibrillation | 2.12 | 2.05 | 2.01 | 1.98 | 1.96 | 0.633 | / |  |
| Race |  |  |  |  |  |  |  |  |
| 1. other |  |  |  |  |  |  |  |  |
| 2. asian | 1.2 | 1.18 | 1.14 | 1.11 | 1.11 | 0.082 | 1.11 | 0.107 |
| 3. black | 1.72 | 1.66 | 1.54 | 1.51 | 1.48 | <0.001 | 1.45 | <0.001 |
| 4. white | 4.03 | 3.95 | 3.45 | 3.32 | 3.31 | <0.001 | 3.26 | <0.001 |
| 5. hipanic | 1.22 | 1.21 | 1.18 | 1.16 | 1.15 | 0.084 | 1.15 | 0.091 |

Note:”/” Represents the removal of corresponding variables in each COX multivariate calculation. The following numbers denote the quantity of COX multivariate calculations executed.In the fourth Cox multivariate analysis, the variance inflation factor (VIF) of urea surpassed 5, suggesting a potential presence of collinearity with creatinine. Given that the p-value for creatinine was greater than 0.05, the variable creatinine was eliminated. In the fifth COX multivariate calculation, all variables exhibited VIF values below 5, so exclusion of variables with p-values less than 0.05.
